# A Novel Class of Cationic and Non-Peptidic Small Molecules as Hits for the Development of Antimicrobial Agents

**DOI:** 10.3390/molecules23071513

**Published:** 2018-06-22

**Authors:** Aranza Jiménez, Pablo García, Sofia de la Puente, Andrés Madrona, María José Camarasa, María-Jesús Pérez-Pérez, José-Carlos Quintela, Francisco García-del Portillo, Ana San-Félix

**Affiliations:** 1 Instituto de Química Médica, Consejo Superior de Investigaciones Científicas (IQM, CSIC), Juan de la Cierva 3, 28006 Madrid, Spain; Aranzaj@hotmail.com (A.J.); sofdelapuen@hotmail.com (S.d.l.P.); andres.madrona@novartis.com (A.M.); mj.camarasa@iqm.csic.es (M.J.C.); mjperez@iqm.csic.es (M.-J.P.-P.); 2 Laboratorio de Patógenos Bacterianos Intracelulares, Centro Nacional de Biotecnología, Consejo Superior de Investigaciones Científicas (CNB, CSIC), Darwin 3, 28049 Madrid, Spain; pgarcia@cnb.csic.es; 3 Natac Biotech S.L., Parque Científico de Madrid, Campus de Cantoblanco, 28049 Madrid, Spain; jcquintela@natac.es

**Keywords:** antimicrobial agents, antibiotic resistance, antimicrobial peptides

## Abstract

Cationic and non-peptide small molecules containing a total of six positive charges arranged on one side and a long aliphatic tail on the other have been synthesized and tested against Gram-positive and Gram-negative bacteria. The positive charges have been contributed by two aminophenol residues. These molecules have showed remarkable antimicrobial activity against Gram-positive bacteria including multidrug-resistant strains. Our structure–activity relationship studies demonstrated the importance of the length and flexibility of the hydrophobic tail for the antimicrobial activity. Importantly, these compounds are non-toxic to eukaryotic cells at the concentration affecting growth in bacteria, reflecting an acceptable margin of safety. The small size and easy synthetic accessibility of our molecules can be of interest for the further development of novel antimicrobials against Gram-positive bacterial pathogens, including multidrug-resistant strains.

## 1. Introduction

At present, microbial resistance to conventional antibiotics is becoming a growing problem that affects, in particular, to hospitals and other health care centers. This crisis has been attributed to the failure to correctly administrate antibiotics or to strictly use them when necessary, to feed farm animals with antibiotics as well as to the lack of investment of the pharmaceutical companies in the development of new antibiotics [1,2,3]. 

According to a recent report of the World Health Organization, if no initiatives are taken the cost of resistance to antibiotics could exceed $100 billion and lead to the premature death of 300 million people in 2050 [4]. Therefore, it is of critical importance to develop new antimicrobial agents in order to substitute or complement currently available antibiotics.

It is well known that many multicellular organisms (animal and plants) produce a variety of peptides, namely antimicrobial peptides (AMPs) and host defense peptides (HDPs), which are effective defensive weapons against a wide range of pathogens including, bacteria, fungi, enveloped viruses, and protozoa [5,6]. 

Naturally occurring AMPs are amphipathic molecules having both hydrophobic residues and hydrophilic positive charges (cationic amino acids), which allow them to bind simultaneously at several sites on the biological membranes [5,6,7,8]. In the particular case of bacteria, the main driving force leading to an efficient binding of these amphipathic molecules is the interaction between the positive charges of AMPs and the negatively charged phospholipid head-groups present on the surface of bacterial membranes [9,10,11,12]. This is followed by interaction of the hydrophobic residues of AMPs with the lipid component of the bacterial membrane, leading to its perturbation, and in certain cases, internalization of the peptide damaging critical intracellular targets [5,6,9]. The ability of AMPs to distinguish between bacterial and mammalian cells is mainly due to the differences in the lipid components of their respective cell membranes. In bacteria, the surface exposed to the outer world is heavily populated by lipids with negatively charged phospholipid head-groups, while the membranes of plant and animals is composed principally of lipids with no net charge [5,9,10,11,12]. 

With respect to conventional antibiotics, AMPs showed several advantages. First, the emergence of resistance against AMPs is less probable than in the case of conventional antibiotics [5,12,13]. In addition, AMPs are able to modulate the immune response though a variety of mechanisms to fight infections [5,6,9,14,15,16]. Moreover, AMPs also show a broad antimicrobial spectrum acting against a variety of pathogens other than bacteria such as viruses, fungi, and protozoa [5,14,15,16]. Despite all of these advantages, AMPs also show several disadvantages that limit their clinical use. Among them, poor bioavailability, potential lability to proteases and high cost of manufacturing [6]. In fact, although some AMPs are currently in clinical trials, there are still few of them available for clinical use—those being polymyxin B, colistin, gramicidin S, daptomycin, and nisin [17,18]. 

Inspired by the antimicrobial activities showed by the natural AMPs, different synthetic mimics have been described over the past few years. Most of them are peptidomimetics but also polymers or oligomers (molecular weight > 1000 Da) [19,20]. With the aim of overcoming the problems associated with the large size of these molecules, more manageable downsized compounds (molecular weight < 1000 Da) have been also synthesized [21,22,23,24,25]. 

**Figure 1:**
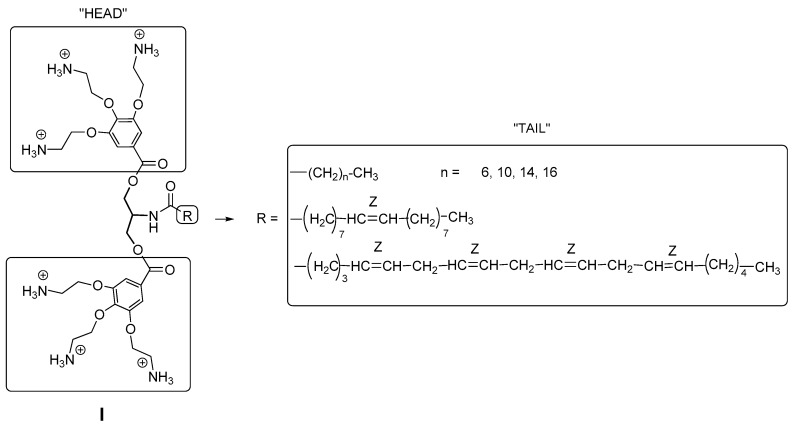
General structure of the synthesized compounds.

Having all of this in mind, we decided to synthesize small molecules (molecular weight < 1000 Da) of general formula **I** (Figure 1) as AMPs mimics. These synthetic molecules bear two aminophenol ‘heads’ on one side and a long aliphatic tail of different length on the other. In the design of these small molecules, some essential structural characteristics of the natural AMPs, such as the simultaneous presence of cationic charges and hydrophobic groups, have been taken into consideration. In our case, the hydrophobicity was provided by the two aromatic rings of the ‘heads’ and the length of the tail, while hydrophilicity by the six positive charges (+6) of the quaternary ammonium groups of the ‘heads’. The length of the alkyl chain, -(CH_2_)*_n_*CH_3_, was varied systematically through variation of the number of methylene groups (*n* = 6 to *n* = 16). Finally, in order to determine the role of the conformational flexibility in the antibacterial activity, analogues in which the aliphatic ‘tail’ has been replaced with an unsaturated chain with one or four double bonds were prepared. 

For these compounds, inhibition of representative drug-sensitive and multidrug-resistant bacterial pathogens has been determined and is herein described.

## 2. Results and Discussion

### 2.1. Chemical Results

The synthesis of the proposed final compounds was achieved in four steps.

**Scheme 1:**
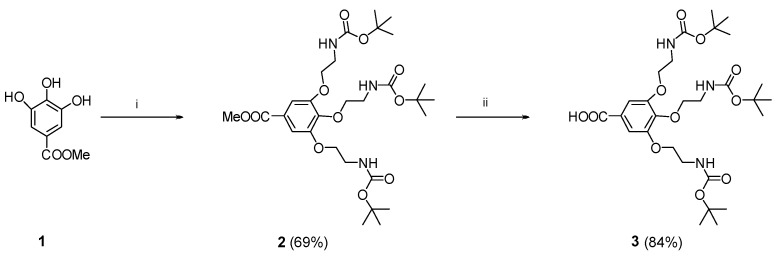
Synthesis of the tris-Boc-aminoethoxy benzoate **3** (aromatic «head»). Reagents and conditions: (i) Br(CH_2_)_2_NHBoc, Cs_2_CO_3_, NaI, acetone, 65 °C (ii) LiOH·H_2_O, then HCl 1M.

The aromatic ‘head’ **3**, containing three Boc-protected amino groups was prepared first (Scheme 1). With this purpose, commercially available methyl 3,4,5-trihydroxybenzoate **1** was treated with 2-(Boc-amino) ethyl bromide in the presence of NaI and Cs_2_CO_3_ (Scheme 1) to afford intermediate **2** (69%). Saponification of the methyl ester present on **2,** using LiOH/H_2_O, afforded compound **3** (84% yield), with a free carboxylic acid on the focal point (Scheme 1). It should be mentioned that the synthesis of **2** has been previously described [26] using potassium carbonate (K_2_CO_3_) as a base. In our hands, this method led to a poor yield of this compound (10%). However, when cesium carbonate and sodium iodide were used, the yield of **2** improved until 69%. 

**Scheme 2:**
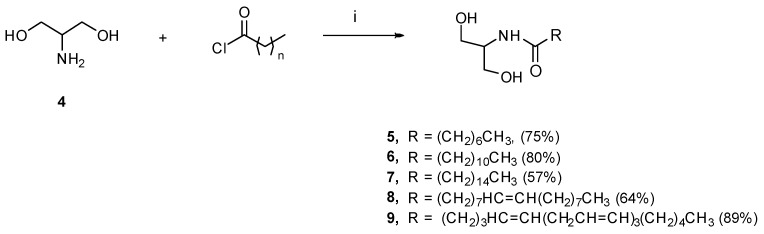
Synthesis of the *N*-acyl serinol derivatives **5**–**9**. Reagents and conditions: (i) MeOH:THF, Et_3_N, −20 °C to room temperature.

Next, reaction of the commercially available 2-amino-1,3-propanediol (serinol) **4** (Scheme 2) with the corresponding acyl chloride, in the presence of trimethylamine at −20 °C, afforded the *N*-acyl intermediates **5**–**9** in moderate to good yields (57–89%). 

**Scheme 3:**
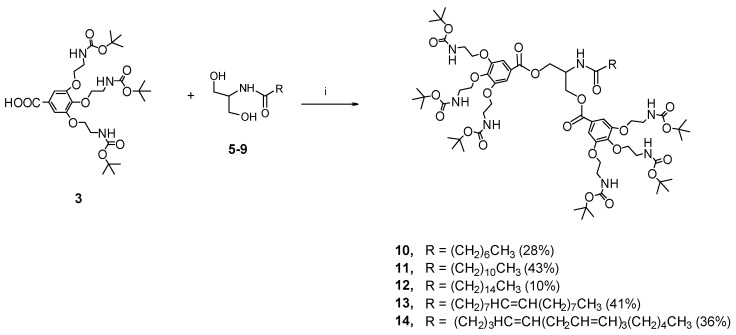
Synthesis of intermediates **10**–**14**. Reagents and conditions: (i) DCC, DMAP, dry dichloromethane.

Subsequent condensation of **5**–**9** with the 2-(Boc-amino) ethyl-protected galloyl acid **3** in the presence of DCC, as coupling reagent and DMAP as base, afforded the Boc protected derivatives **10**–**14** in low to moderate yields (10–43%) (Scheme 3).

**Scheme 4:**
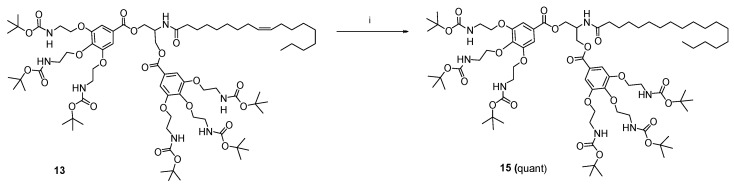
Synthesis of the fully saturated derivative **15**. Reagents and conditions: (i) H_2_, Pd/C (10%), EtOAc, rt.

Catalytic hydrogenation of **13** at atmospheric pressure, in the presence of 10% Pd/C in ethyl acetate, afforded the saturated derivative **15** in quantitative yield (Scheme 4).

**Scheme 5:**
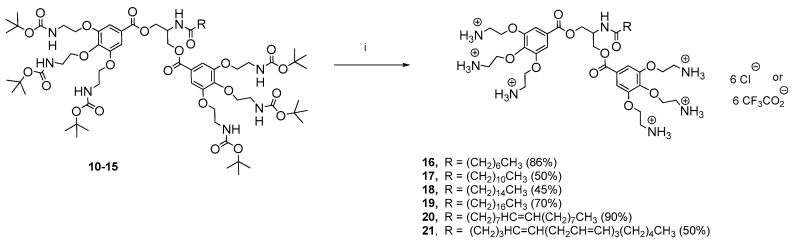
Synthesis of the deprotected final derivatives **16**–**21**. Reagents and conditions: (i) TFA or HCl, dichloromethane, rt.

Finally, treatment of the Boc-protected derivatives **10**–**15** with TFA or HCl gave the deprotected final compounds **16**–**21** (45–90%) as their corresponding salts (Scheme 5).

### 2.2. Biological Results

#### 2.2.1. Antibacterial Activity

The antimicrobial activities of the new aminophenol derivatives (**16**–**21**) were tested against representative drug-sensitive and multidrug-resistant bacterial pathogens. As drug-sensitive bacteria *Salmonella enterica* serovar Typhimurium SV5015 (*S.* Typhimurium SV5015) (Gram-negative), *Listeria monocytogene*s EGD-e (*L. monocytogenes* EGD-e) (Gram-positive), and *Staphylococcus aureus* Newman (*S. aureus* Newman) (Gram-positive) have been chosen. As drug-resistant bacteria *Staphylococcus aureus* SC-1 (*S. aureus* SC-1) and *Staphylococcus aureus* USA-300 *(S. aureus* USA-300) have been used.

**Figure 2:**
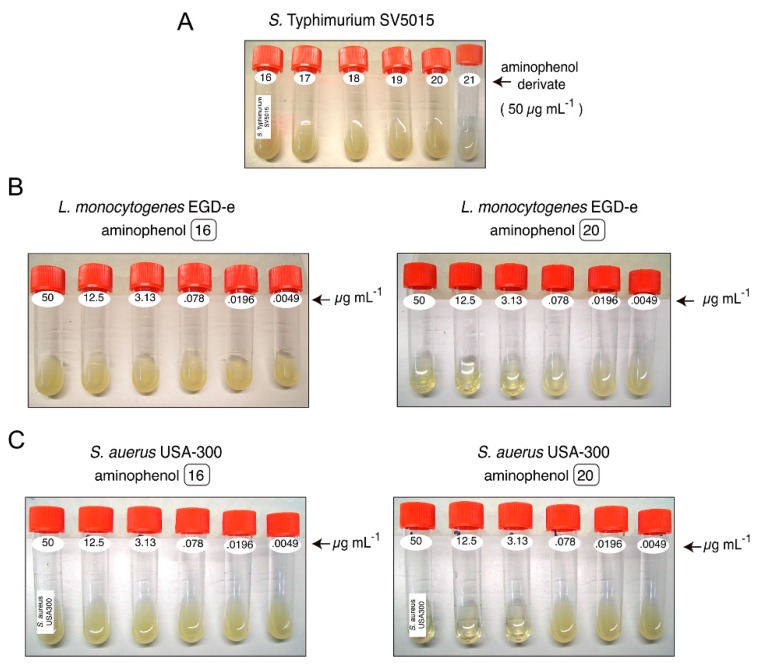
Antimicrobial assays in liquid culture using different aminophenol compounds and bacterial species. Bacteria were cultured in media containing the aminophenol for 18 h overnight culture. (**A**) Lack of effect of the test compounds (50 µg mL^−1^) in the Gram-negative bacterial pathogen *S.* Typhimurium strain SV5015; (**B**) Differential effect of aminophenol compounds **16** and **20** in the Gram-positive bacterial pathogen *L. monocytogenes* strain EGD-e. Note the lack of effect of compound **16** and the inhibitory effect of compound **20**. Similar results as for compound **20** were obtained for compounds **17**, **18**, **19**, and **21** (not shown); (**C**) Differential effect of aminophenol compounds **16** and **20** in the multidrug resistant clinical isolate *S. aureus* USA-300 strain. Note the lack of effect of compound **16** and the inhibitory effect of compound **20**. Similar results as for compound **20** were obtained for compounds **17**, **18**, **19**, and **21** (not shown). The compounds **17**, **18**, **19**, **20**, and **21** also displayed inhibitory capacity against *S. aureus* strains Newman and SC-1 (not shown, see text for details).

Initial assays performed in liquid culture showed that none of these compounds **16**–**21** proved inhibitory against the Gram-negative bacteria *S.* Typhimurium SV5015 up to 50 µg mL^−1^ (Figure 2A). However, with the exception of **16**, the rest of compounds **17**–**21** showed inhibitory effects against the Gram-positive bacteria *L. monocytogenes* EGD-e (only **20**, as representative of the active compounds, is showed in Figure 2B). Interestingly, **17**–**21** also showed inhibitory effects against the multidrug-resistant bacteria *S. aureus* USA-300 (only **20**, as representative of the active compounds, is showed in Figure 2C).

Next, the minimal inhibitory concentrations (MIC) of **16**–**21** have been determined. In this experiment, kanamycin—a conventional aminoglycoside antibiotic that inhibits the synthesis of bacterial proteins—was used as control [27]. Table 1 summarizes the results of this evaluation.

As it was shown in Table 1, the *N*-octanoyl derivative **16**, with an acyl chain containing only 6 methylenes, did not show significant activity up to 50 µg mL^−1^ against any of the drug-sensitive bacteria (*Salmonella enterica* serovar Typhimurium strain SV5015, *Listeria monocytogenes* strain EGD-e, and *Staphylococcus aureus* strain Newman)*.* The *N*-dodecanoyl analogue, compound **17,** with 10 methylenes, displayed improved antibacterial activity against *L. monocytogenes* EGD-e and *S. aureus* Newman (MIC: 12.5 and 50 µg mL^−1^, respectively)*.* Further increase in the length of the aliphatic chain (14 methylenes) yielded compound **18** (*N*-hexadecanoyl analogue) that displayed much improved antibacterial activity against *L. monocytogenes* EGD-e and *S. aureus* Newman (MIC: 3.13 and 12.5 µg mL^−1^, respectively). However, the highest long chain analogue **19** (*N*-octadecanoyl analogue, 16 methylenes) did not display any significant change in activity with respect to **18**, indicating that the additional two methylene groups present in the aliphatic chain of **19** are superfluous. 

The effect of increasing the rigidity of the molecule in the antibacterial activity was analyzed with compounds **20** and **21**, with one and four double bonds, respectively, in the aliphatic chain. The unsaturated analogue **20**, with only one double bond in the aliphatic chain, was found to be as active against *L. monocytogenes* EGD-e and *S. aureus* Newman as the saturated counterparts **18** and **19**, whereas compound **21**, containing four double bonds was less active, suggesting that an excessive conformational rigidity in the aliphatic chain is detrimental for the antibacterial activity. 

The new aminophenol derivatives **16**–**21** were also tested for their in vitro inhibitory effects on clinically isolated multidrug-resistant (MDR) bacteria such as *S. aureus* strains SC-1 and USA-300 [28]. 

Compounds **17** and **21** only showed moderate activity (MIC: 50 µg mL^−1^) against the drug-resistant strain *S. aureus* SC-1, while they showed significant activity (MIC: 12.5 µg mL^−1^) against the drug-resistant strain *S. aureus* USA-300. Just the opposite was observed for compound **19,** which displayed moderate activity (MIC: 50 µg mL^−1^) against *S. aureus* USA-300 strain and significant (MIC: 12.5 µg mL^−1^) against *S. aureus* SC-1. Interestingly, the activity showed by compounds **18** and **20** against both resistant strains was remarkable. It contrasted with the lack of activity showed by the conventional antibiotic kanamycin, which was used as control. Similar to the results found for the drug-sensitive strains, compound **16**, with the shortest aliphatic chain (octanoyl chain), did not show significant activity up to 50 µg mL^−1^ against any of the multidrug-resistant bacteria. 

The saturated compound **18**, with a long aliphatic chain (C16) and **20** with a long chain (C18) containing only one unsaturation, were therefore the most potent compounds of this series against all the tested bacteria strains (drug-sensitive and multidrug-resistant). 

It should be noted that the activity showed by **18** and **20** against Gram-positive drug-sensitive bacteria is very similar to that of the conventional antibiotic used as control, kanamycin. Moreover, **18** and **20** resulted active against drug-resistant bacterial strains for which kanamycin was inactive. 

In summary, the structure–activity relationship (SAR) studies reveal the significance of our design for achieving Gram-positive antimicrobial activity, especially against drug-resistant bacterial strains. Our molecules, that display the most important characteristic of most AMPs, like cationic facial amphiphilicity and positive charges, have several advantages over the natural counterparts. Among them, non-peptidic character, low-cost synthesis, and possibility to fine-tuning their potency and safety. Based on that, they can be useful hits for the further design of novel antimicrobial agents against Gram-positive bacteria.

#### 2.2.2. Antimicrobial Kinetics 

Next, the effect of the newly synthesized compounds over the bacterial growth in function of time was determined. Bacterial growth (cells per volume unit), estimated from the turbidity of the culture, was measured using a spectrophotometer at a wavelength of 600 nm and it was represented as optical density (OD_600_). 

**Figure 3:**
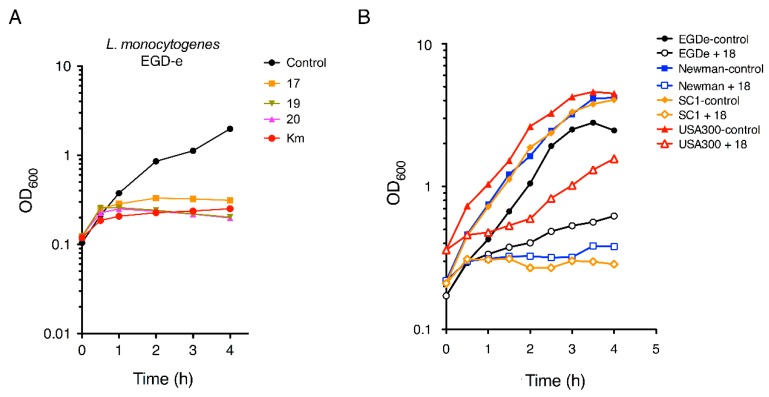
The aminophenol compounds arrest efficiently growth of *L. monocytogenes* and *S. aureus*. (**A**) Growth curve of a culture of *L. monocytogenes* strain EGD-e exposed to the indicated compounds. Time 0 in the graphic corresponds to the time the compound was added to the culture (OD600 ~0.15–0.20). All antimicrobial was used at four-fold of their respective MIC value for each of the indicated bacterial strains (see Table 1); (**B**) Growth curves of *L. monocytogenes* strain EGD-e and *S. aureus* cultures exposed to the amino phenol **18**. Amino phenol **18** was used at four-fold of its respective MIC value for each of the indicated bacterial strains (see Table 1).

First, we monitored the growth over time of the drug-sensitive bacteria *L. monocytogenes* EGD-e in the presence of compounds **17**, **19**, and **20**, used at a concentration that was four-fold higher than their respective MICs (4 MIC) (see Table 1). The aminoglycoside kanamycin at 30 µg mL^−1^ was used as relevant control since it rapidly arrests growth in actively proliferating bacteria [27]. As it was shown in Figure 3A the curves for compounds **17**, **19**, and **20** are very similar to those of the kanamycin. Thus, this experiment clearly shows that the treatment with aminophenols **17**, **19**, and **20** resulted in rapid arrest of bacteria growth (Figure 3A) and, consequently, these compounds behave as potent antimicrobials. 

Next, the growth over time of the drug-sensitive bacteria *L. monocytogenes* EGD-e and *S. aureus* Newman, together with the multidrug-resistant bacteria *S. aureus* strains SC1 and USA300 was analyzed in the presence of the most potent aminophenol derivative **18** (4 MIC) (Figure 3B). Our results showed that **18** is able of arresting the growth of the four studied bacterial strains. Apparently, *S. aureus* Newman (drug-sensitive) and *S. aureus* SC-1 (multidrug-resistant) strains were more sensitive to the action of **18** than the other bacterial strains (Figure 3B). 

#### 2.2.3. Cytotoxicity Evaluation

**Figure 4:**
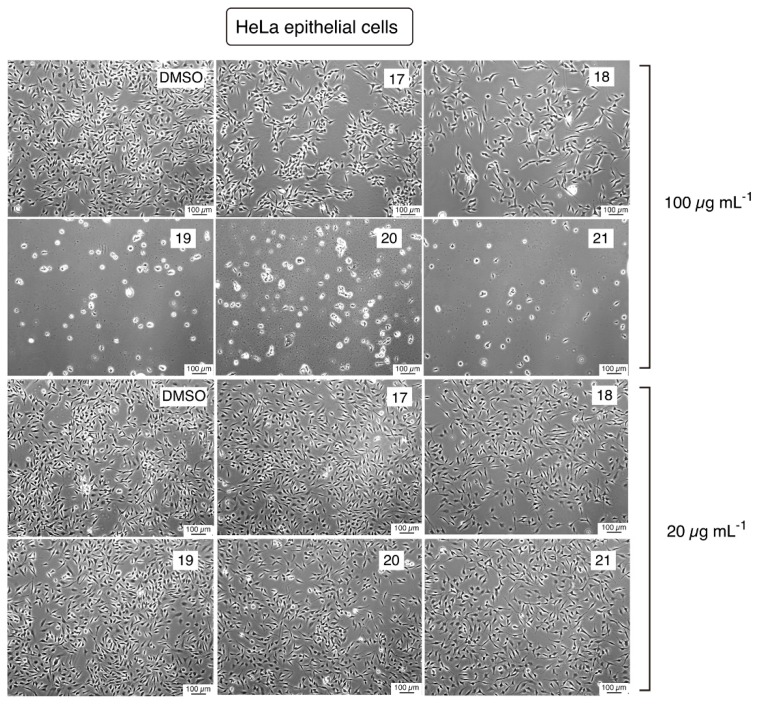
Toxicity assay of the series of compounds **17** to **21** in human HeLa epithelial cells. The cell culture was exposed to the different compounds for 18 h at different concentrations (only 100 µg mL^−1^ or 20 µg mL^−1^ was indicated). Cells were fixed and imaged in an inverted microscopy as described in Section 4.

**Figure 5:**
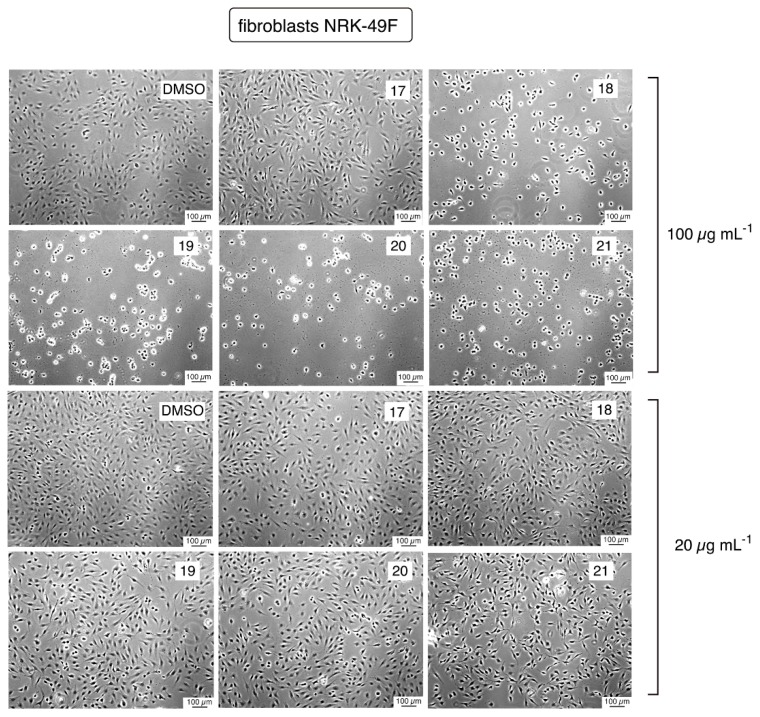
Toxicity assay of the series of compounds **17**–**21** in rat fibroblasts NRK-49F. The cell culture was exposed to the different compounds for 18 h at different concentrations (only two concentrations 100 µg mL^−1^ or 20 µg mL^−1^ are indicated). Cells were fixed and imaged in an inverted microscopy as described in Materials and Methods. In the case of compound **17,** toxicity in NRK-49F fibroblasts was not observed at the highest concentration tested (100 µg mL^−1^).

Finally, the cytotoxic effects of the aminophenol derivatives **17**–**21** were determined in two different eukaryotic cell types, HeLa epithelial cells (Figure 4) and NRK-49F fibroblasts (Figure 5).

Different concentrations of the aminophenol compounds **17**–**21** were used in these experiments. After an overnight exposure to the compounds, toxicity—denoted by detachment of cells and drastic morphological changes—was evident only at 100 µg mL^−1^ while no toxicity was found at lower concentrations. Importantly, the highest concentration (100 µg mL^−1^) was 8-fold or 32-fold higher than the MIC value observed for **17**–**21** in the antimicrobial assays (see Table 1) reflecting an acceptable margin of safety. 

## 3. Conclusions

In this study, we have developed an efficient strategy for the synthesis of novel aminophenol derivatives **17**–**21**. All of these compounds have the same hydrophilic portion consisting in two cationic ‘heads’ with three positive charges each (six positive charges in total) and a lipophilic ‘tail’ of different length and conformational flexibility. These compounds were synthesized in four simple synthetic steps.

The synthesized compounds showed antimicrobial activity against different Gram-positive bacteria (*L. monocytogenes* and *S. aureus*) while probed to be inactive against Gram negative bacteria (*S. Typhimurium*). Interestingly **17**–**21** also showed inhibitory effects against Gram-positive multidrug-resistant strains such as *S. aureus* SC-1 and *S. aureus* USA-300. 

In this respect, it should be noted that defined AMPs have been reported to be only active against Gram-positive bacteria while others are not selective and showed broad activity against both Gram-negative and Gram-positive bacteria [29,30,31]. Despite their mode of action not being fully understood and the basis for their selectivity being unknown, many studies reveal the importance of the membrane components of bacteria—lipid content in particular—in the activity and selectivity of these molecules [29,30,31]. 

These precedents support the idea of a mechanism of action for the compounds here described directly related to a defined structure in the bacterial cell envelope. Further studies are required to decipher their mode of action and selectivity towards microbial cells. 

Presence of long chains (C16 or C18) is an important structural requirement for antimicrobial activity. Introduction of one double bond in a long alkyl chain (C18) renders molecules that also showed good antibacterial activity while the incorporation of four double bonds led to much less active molecules. This finding emphasized the importance of the length and flexibility of the hydrophobic alkyl portion (‘tail’) for the antibacterial activity. 

Compounds **18** (C16) and **19** (C18) with saturated long chains, together with **20,** that incorporates one double bond in its long chain (C18), were found to be the most potent antibacterial agents among this series. These compounds are non-toxic to eukaryotic cells at the concentration affecting growth in bacteria, reflecting an acceptable margin of safety.

Taken together, these data proved that the newly synthesized aminophenol compounds can be promising hits for the further development of antimicrobial agents against Gram-positive bacteria (drug-sensitive and multidrug-resistant). However, further research is needed to elucidate the pharmacophore group(s) and their mechanism of action. 

## 4. Materials and Methods

### 4.1. Synthesis

Commercial reagents and solvents were used as received from the suppliers without further purification unless otherwise stated. The solvents used in some reactions were dried prior use. DMF dry was commercially available (Sigma-Aldrich Quimica SL, Madrid, Spain).

Analytical thin-layer chromatography (TLC) was performed on aluminum plates pre-coated with silica gel 60 (F_254_, 0.25 mm). Products were visualized using an ultraviolet lamp (254 and 365 nm) or by heating on a hot plate (approx. 200 °C), directly or after treatment with a 5% solution of phosphomolybdic acid or vanillin in ethanol. 

The compounds were purified by: (a) High Performance Flash Chromatography (HPFC) with a system “Isolera One” (Biotage, Uppsala, Sweden) in reverse phase using water/acetonitrile (100:0 to 0:100) as eluent; (b) flash column chromatography on silica gel (60 Merck 230–400 mesh); and (c) preparative centrifugal circular thin layer chromatography (CCTLC) on a chromatotron^®^ (Kiesegel 60 PF254 gipshaltig, Merck, Dramstand, Germany) layer thickness 1 mm, flow rate 2–4 mL/min.

For HPLC analysis, an Agilent Technologies 1120 Compact LC with a reverse phase column ACE 5 C18-300 (4.6 × 150 mm, 3.5 µm) equipped with a PDA (photodiode array) detector Waters 2996 was used. Acetonitrile was used as mobile phase A, and water with 0.05% of TFA was used as mobile phase B at a flow rate of 1 mL min^−1^. All retention times are quoted in minutes and the gradients are specified for each compound in the experimental data. 

For high resolution mass spectrometry (HRMS) was used an Agilent 6520 Accurate Mass QTOF (quadrupole time-of-flight) coupled with LC/MS using an electrospray interface (ESI) working in the positive-ion (ESI^+^) and negative-ion (ESI^−^) mode.

NMR spectra (^1^H and ^13^C NMR) were recorded on a Varian UNIT INOVA-300 (300 MHz) (now Agilent, Santa Clara, CA, USA), Bruker AVANCE 300 (300 and 75 MHz) (Bruker, Billerica, MA, USA), Varian INOVA-400 (400 and 100 MHz) (now Agilent, Santa Clara, CA, USA), Varian MERCURY-400 (now Agilent, Santa Clara, CA, USA) (400 and 100 MHz), and Varian-500 (now Agilent, Santa Clara, CA, USA) (500 and 125 MHz) spectrometers, using DMSO-*d*_6_ and CDCl_3_ as solvents. Chemical shift (δ) values are reported in parts per million (ppm) relative to tetramethylsilane (TMS) in ^1^H and CDCl_3_ (δ = 77.0) in ^13^C NMR. Coupling constant (*J* values) are reported in hertz (Hz) and multiplicities of signals are indicated by the following symbol: s (singlet), d (doublet), t (triplet), q (quadruplet), m (multiplet), and bs (broad singlet). Some two-dimensional spectra (COSY, HSQC, and HMBC) were performed to identify the structure. 

Final compounds were lyophilized using a Telstar 6–80 systhem.

#### 4.1.1. Methyl 3,4,5-tris[2-(Boc-amino)-1-ethoxy]benzoate (**2**)

A mixture of methyl 3,4,5-trihydroxybenzoate **1** (250 mg, 1.35 mmol), Cs_2_CO_3_ (1.75 g, 5.4 mmol), NaI (203 mg, 1.35 mmol) and 2-(Boc-amino)ethyl bromide (1.2 g, 5.4 mmol) in acetone (10 mL) was refluxed overnight at 65 °C and then evaporated to dryness. The residue was dissolved in ethyl acetate (20 mL) and washed with aqueous solutions of citric acid (10%) (3 × 20 mL) and brine (2 × 20 mL). The organic phase was dried over anhydrous Na_2_SO_4,_ filtered and evaporated to dryness. The residue was purified on a Biotage HPFC (high performance flash chromotography) purification system in a reverse phase using water/acetonitrile (100:0 to 0:100) to afford 576 mg (69%) of **2** as a yellow foam. ^1^H NMR (400 MHz, DMSO-*d*_6_) δ 7.20 (s, 2H, Ar), 6.96 (t, *J* = 5.7 Hz, 2H, NHCO), 6.63 (t, *J* = 5.7 Hz, 1H, NHCO), 4.01 (t, *J* = 5.7 Hz, 4H, CH_2_O), 3.96 (t, *J* = 5.9 Hz, 2H, CH_2_O), 3.83 (s, 3H, CH_3_O), 3.35 (m, 4H, CH_2_NH), 3.22 (m, 2H, CH_2_NH), 1.35 (s, 18H, Boc), 1.36 (s, 9H, Boc). HPLC (t_R_) [gradient: A:B, 10–100% of A in 10 min]: 9.54 min.

#### 4.1.2. 3,4,5-tris[2-(Boc-amino-1-ethoxy]benzoate (**3**) 

To a solution at 0 °C containing **2** (333 mg, 0.54 mmol) in THF (15 mL), a second solution of LiOH·H_2_O (45 mg, 1.08 mmol) in water (7 mL) was added. The mixture was stirred at room temperature overnight. Then, an aqueous solution of 1N hydrochloric acid was added to reach pH = 3–4, and the mixture was evaporated to dryness. The residue was dissolved in ethyl acetate (20 mL) and washed with H_2_O (3 × 20 mL). The organic phase was dried over anhydrous Na_2_SO_4_, filtered and evaporated to dryness to afford 272 mg (84%) of **3** as an amorphous white solid. ^1^H NMR (300 MHz, DMSO-*d*_6_) δ 7.21 (s, 2H, Ar), 6.77 (br s, 2H, NHCO), 6.45 (br s, 1H, NHCO), 4.01 (m, 6H, CH_2_O), 3.35 (m, 4H, CH_2_NH), 3.23 (m, 2H, CH_2_NH), 1.37 (s, 18H, Boc), 1.36 (s, 9H, Boc). HPLC (t_R_) [gradient: A:B, 10–100% of A in 10 min]: 5.32 min.

#### 4.1.3. General Procedure for the Synthesis of the *N*-acyl Serinol Derivatives **5**–**9**

A stirred solution of 2-amino-1,3-propanediol (serinol) **4** (1 eq) and triethylamine (TEA) in MeOH (10 mL) was cooled at −20 °C (carbon dioxide snow) and added dropwise a solution of the corresponding acyl chloride (1.1 eq) in THF (5 mL). The reaction mixture was allowed to warm to room temperature and stirred overnight. Then it was poured into brine and extracted with dichloromethane (3 × 10 mL). The combined organic phases were washed with brine, dried over MgSO_4_ and concentrated under reduced pressure. The residue was purified by flash column chromatography on silica gel using as eluent EtOAc/MeOH (20:1 to 7:1) to provide the corresponding *N*-acyl serinol derivatives.

#### 4.1.4. *N*-octanoyl Serinol (**5**)

According to the general procedure serinol **4** (150 mg, 1.64 mmol) was treated with octyl chloride (1.81 mmol, 0.3 mL) and TEA (0.4 mL) to give 267 mg (75%) of **5** as an amorphous white solid. ^1^H NMR (400 MHz, DMSO-*d*_6_) δ 7.40 (d, *J* = 8.1 Hz, 1H, NHCO), 4.55 (t, *J* = 5.5 Hz, 2H, OH), 3.69 (m, 1H, *CH*NH), 3.37 (t, *J* = 5.6 Hz, 4H, *CH_2_*OH), 2.06 (t, *J* = 7.4 Hz, 2H, CH_2_CO), 1.46 (m, 2H, CH_2_), 1.22 (m, 8H, CH_2_), 0.86 (t, *J* = 6.8 Hz, 3H, CH_3_). 

#### 4.1.5. *N*-dodecanoyl Odecanoyl Serinol (**6**)

According to the general procedure serinol **4** (200 mg, 2.19 mmol) was treated with dodecanoyl chloride (625 mg, 2.8 mmol) and TEA (0.6 mL) to give 478 mg, (80%) of **6** as an amorphous white solid. Spectroscopic data of this compound are consistent with those found in the literature [32]. 

#### 4.1.6. *N*-hexadecanoyl Exadecanoyl Serinol (**7**)

According to the general procedure serinol **4** (150 mg, 1.64 mmol) was treated with hexadecanoyl chloride (1.81 mmol, 0.5 mL) and TEA (0.4 mL) to give 309 mg (57%) of **7** as an amorphous white solid. ^1^H NMR (400 MHz, DMSO-*d*_6_) δ 7.39 (d, *J* = 8.1 Hz, 1H, NHCO), 4.55 (t, *J* = 5.5 Hz, 2H, OH), 3.66 (m, 1H, CHNH), 3.35 (t, *J* = 5.6 Hz, 4H, CH_2_OH), 2.03 (t, *J* = 7.4 Hz, 2H, CH_2_CO), 1.43 (m, 2H, CH_2_), 1.21 (m, 24H, CH_2_), 0.83 (t, *J* = 6.6 Hz, 3H, CH_3_). 

#### 4.1.7. *N*-oleoyl Serinol (**8**)

According to the general procedure serinol **4** (150 mg, 1.64 mmol) was treated with 9-octadecenoyl chloride (oleoyl chloride) (541.6 mg, 0.6 mL) and TEA (0.4 mL) to give 375 mg (64%) of **8** as an amorphous white solid. Spectroscopic data of this compound are consistent with those found in the literature [33]. 

#### 4.1.8. *N*-arachidonoyl Serinol (**9**) 

A solution of *Z,Z,Z,Z*-5,8,11,14-eicosatetraenoic acid (arachidonic acid) (510 mg, 1.5 mmol) and thionyl chloride (87 mmol, 6.3 mL) in dry dichloromethane (10 mL) was stirred at room temperature under argon atmosphere until the reaction is complete. The solvent was evaporated to dryness and the residue was used immediately for the next step.

According to the general procedure, serinol **4** (49 mg, 0.54 mmol) was treated with the above mentioned arachidonic chloride (193 mg, 0.6 mmol) and TEA (0.1 mL) to give 182 mg (89%) of **9** as an amorphous white solid. ^1^H NMR (400 MHz, DMSO-*d*_6_) δ 7.42 (d, *J* = 8.1 Hz, 1H, NHCO), 5.34 (m, 8H, CH=), 4.54 (t, *J* = 5.6 Hz, 2H, OH), 3.68 (m, 1H, CHNH), 3.38 (t, *J* = 5.4 Hz, 4H, CH_2_OH), 2.79 (m, 6H, CH_2_CH=), 2.08 (t, *J* = 7.5 Hz, 2H, CH_2_CO), 2.02 (m, 4H, CH_2_CH=), 1.53 (m, 2H, CH_2_), 1.40–1.16 (m, 6H, CH_2_), 0.85 (t, *J* = 6.7 Hz, 3H, CH_3_). 

#### 4.1.9. General Coupling Procedure for the Synthesis of the Boc Protected Intermediates **10**–**14**

A solution of the Boc protected gallic acid derivative **3** (2.2 eq), DMAP (1.6 eq), and DCC (3.2 eq) in dry CH_2_Cl_2_ (7.5 mL) was stirred at room temperature for 15 min and then added dropwise to a second solution, also stirred for 15 min, containing the corresponding serinol derivate **5**–**9** (1 eq) and DMAP (1.6 eq) in dry CH_2_Cl_2_ (7.5 mL). To dissolve all components, an extra amount of DMF (2 mL) were added. The solution was stirred at 30 °C for 24 h and then evaporated to dryness. The residue was dissolved in ethyl acetate (20 mL). Urea by-product was filtered and the organic residue was evaporated. This process was repeated several times until no precipitation was observed. The residue was purified on a Biotage HPFC purification system in a reverse phase using water:acetonitrile (100:0 to 0:100) to give the corresponding intermediate. Note that all the solid reactives are pre-dried overnight by storage inside a vacuum desiccator with a drying agent like phosphorous pentoxide.

#### 4.1.10. Bis-*O*-[3,4,5-tris(2-*N*-Boc-amino-1-ethoxy)benzoyl]-*N*-octanoyl Serinol (**10**)

Following the general procedure, **5** (25 mg, 0.1 mmol) was treated with **3** (203.1 mg, 0.34 mmol) to afford 47.3 mg (28%) of **10** as an amorphous white solid. ^1^H NMR (400 MHz, DMSO-*d*_6_) δ 8.08 (d, *J* = 8.4 Hz, 1H, NHCO), 7.24 (s, 4H, Ar), 6.98 (t, *J* = 5.7 Hz, 4H, NHCO), 6.64 (m, 2H, NHCO), 4.58 (m, 1H, CHNH), 4.40 (dd, *J* = 11.2, 5.1 Hz, 2H, CH_2_O), 4.30 (dd, *J* = 11.1, 6.8 Hz, 2H, CH_2_O), 3.99 (m, 12H, CH_2_O), 3.36 (m, 8H, CH_2_N), 3.23 (m, 4H, CH_2_N), 2.09 (t, *J* = 7.3 Hz, 2H, CH_2_CO), 1.45 (t, *J* = 7.2 Hz, 2H, CH_2_), 1.37 (s, 54H, Boc), 1.20-1.01 (m, 8H, CH_2_), 0.77 (t, *J* = 7.1 Hz, 3H, CH_3_). HPLC (t_R_) [isocratic of acetonitrile A:B, 0–100%]: 2.3 min. 

#### 4.1.11. Bis-*O*-[3,4,5-tris(2-*N*-Boc-amino-1-ethoxy)benzoyl]-*N*-dodecanoyl Serinol (**11**) 

Following the general procedure, **6** (50 mg, 0.2 mmol) was treated with **3** (240 mg, 0.4 mmol) to afford 112.5 mg (43%) of **11** as an amorphous white solid. ^1^H NMR (300 MHz, DMSO-*d*_6_) δ 8.11 (d, *J* = 8.3 Hz, 1H, NHCO), 7.23 (s, 4H, Ar), 7.01 (t, *J* = 5.5 Hz, 4H, NHCO), 6.68 (br s, 2H, NHCO), 4.57 (m, 1H, *CH*NH), 4.37 (m, 2H, CH_2_O), 4.30 (m, 2H, CH_2_O), 3.98 (m, 12H, CH_2_O), 3.36 (m, 8H, CH_2_N), 3.21 (m, 4H, CH_2_N), 2.09 (t, *J* = 7.2 Hz, 2H, CH_2_CO), 1.36 (m, 56H, CH_2_ and Boc), 1.26–1.03 (m, 16H, CH_2_), 0.84 (t, *J* = 6.5 Hz, 3H, CH_3_). HPLC (t_R_) [isocratic of acetonitrile A:B, 0–100%]: 2.7 min. 

#### 4.1.12. Bis-*O*-[3,4,5-tris(2-*N*-Boc-amino-1-ethoxy)benzoyl]-*N*-hexadecanoyl Serinol (**12**) 

Following the general procedure, **7** (25 mg, 0.07 mmol) was treated with **3** (127 mg, 0.21 mmol) to afford 11.8 mg (10%) of **12** as an amorphous white solid. ^1^H NMR (300 MHz, DMSO-*d*_6_) δ 8.11 (d, *J* = 8.3 Hz, 1H, NHCO), 7.23 (s, 4H, Ar), 7.00 (t, *J* = 5.5 Hz, 4H, NHCO), 6.66 (t, *J* = 5.6 Hz, 2H, NHCO), 4.56 (m, 1H, *CH*NH), 4.36 (m, 2H, CH_2_O), 4.27 (m, 2H, CH_2_O), 3.94 (m, 12H, CH_2_O), 3.33 (m, 8H, CH_2_N), 3.20 (m, 4H, CH_2_N), 2.06 (t, *J* = 7.2 Hz, 2H, CH_2_CO), 1.36 (m, 56H, CH_2_ and Boc), 1.25–1.05 (m, 24H, CH_2_), 0.84 (t, *J* = 6.3 Hz, 3H, CH_3_). HPLC (t_R_) [isocratic of acetonitrile A:B, 0–100%]: 3.5 min.

#### 4.1.13. Bis-*O*-[3,4,5-tris(2-*N*-Boc-amino-1-ethoxy)benzoyl]-*N*-oleoyl Serinol (**13**) 

Following the general procedure, **8** (50 mg, 0.14 mmol) was treated with **3** (236.1 mg, 0.39 mmol) to afford 87 mg (41%) of **13** as an amorphous white solid. ^1^H NMR (300 MHz, DMSO-*d*_6_) δ 8.10 (d, *J* = 8.5 Hz, 1H, NHCO), 7.24 (s, 4H, Ar), 7.00 (t, *J* = 5.7 Hz, 4H, NHCO), 6.65 (d, *J* = 5.8 Hz, 2H, NHCO), 5.28 (dd, *J* = 4.0, 1.5 Hz, 2H, CH=), 4.58 (m, 1H, *CH*NH), 4.41 (dd, *J* = 10.9, 4.9 Hz, 2H, CH_2_O), 4.30 (dd, *J* = 11.1, 6.8 Hz, 2H, CH_2_O), 3.99 (m, 12H, CH_2_O), 3.33 (m, 8H, CH_2_N), 3.22 (m, 4H, CH_2_N), 2.10 (t, *J* = 7.4 Hz, 2H, CH_2_CO), 1.93 (m, 4H, CH_2_CH=), 1.37 (m, 56H, CH_2_ and Boc), 1.29–1.12 (m, 20H, CH_2_), 0.84 (t, *J* = 6.7 Hz, 3H, CH_3_). HPLC (t_R_) [isocratic of acetonitrile A:B, 0–100%]: 3.5 min.

#### 4.1.14. Bis-*O*-[3,4,5-tris(2-*N*-Boc-amino-1-ethoxy)benzoyl]-*N*-arachidonoyl Serinol (**14**) 

Following the general procedure, **9** (78 mg, 0.20 mmol) was treated with **3** (308 mg, 0.51 mmol) to afford 113 mg (36%) of **14** as an amorphous white solid. ^1^H NMR (400 MHz, DMSO-*d*_6_) δ 8.12 (d, *J* = 8.3 Hz, 1H, NHCO), 7.24 (s, 4H, Ar), 6.98 (t, *J* = 5.8 Hz, 4H, NHCO), 6.64 (d, *J* = 6.6 Hz, 2H, NHCO), 5.30 (m, 8H, CH=), 4.56 (m, 1H, CHNH), 4.40 (t, *J* = 6.4 Hz, 2H, CH_2_O), 4.30 (dd, *J =* 11.1, 6.6 Hz, 2H, CH_2_O), 3.98 (m, 12H, CH_2_O), 3.33 (m, 8H, CH_2_N), 3.23 (m, 4H, CH_2_N), 2.73 (m, 4H, CH_2_CH=), 2.70 (m, 2H, CH_2_CH=), 2.11 (m, 2H, CH_2_CO), 2.00 (m, 4H, CH_2_CH=), 1.50 (m, 2H, CH_2_), 1.37 (s, 54H, Boc), 1.25 (m, 6H, CH_2_), 0.83 (t, *J* = 6.7 Hz, 3H, CH_3_). HPLC (t_R_) [isocratic of acetonitrile A:B, 0–100%]: 3.8 min.

#### 4.1.15. Bis-*O*-[3,4,5-tris(2-*N*-Boc-amino-1-ethoxy)benzoyl]-*N*-octadecanoyl Serinol (**15**) 

A solution of **13** (114 mg, 7.05 mmol) in ethyl acetate containing 30% wt. of 10% Pd/C was hydrogenated at 30 °C for 4–6 h under atmospheric pressure using a reaction balloon filled with hydrogen gas and a glass flask as the reaction vessel. The Pd/C was filtered through Whatman^®^ filter paper 42 and the solvent was removed under reduced pressure to give 110.3 mg (quant) of the title compound as an amorphous white solid. ^1^H NMR (400 MHz, DMSO-*d*_6_) δ 8.08 (d, *J* = 8.3 Hz, 1H, NHCO), 7.24 (s, 4H, Ar), 6.98 (m, 4H, NHCO), 6.63 (m, 2H, NHCO), 4.57 (m, 1H, CHNH), 4.40 (dd, *J* = 11.2, 5.1 Hz, 2H, CH_2_O), 4.30 (dd, *J* = 11.1, 6.8 Hz, 2H, CH_2_O), 3.99 (m, 12H, CH_2_O), 3.32 (m, 8H, CH_2_N), 3.21 (m, 4H, CH_2_N), 2.08 (t, *J* = 7.3 Hz, 1H, CH_2_CO), 1.48 (m, 2H, CH_2_), 1.38 (s, 54H, Boc), 1.27–1.01 (m, 28H, CH_2_), 0.85 (t, *J* = 6.8 Hz, 3H, CH_3_). HPLC (t_R_) [isocratic of acetonitrile A:B, 0–100%]: 4.5 min.

#### 4.1.16. Synthesis of the Deprotected Serinol Derivatives **16**–**21**

To a solution containing the corresponding Boc protected derivative (**10**–**15**) in CH_2_Cl_2_ (10 mL), TFA was added. After stirring at room temperature overnight the solution was evaporated to dryness and co-evaporated successively with CH_2_Cl_2_ and MeOH. The residue was lyophilized to afford the final products.

#### 4.1.17. Bis-*O*-[3,4,5-tris(2-ammonium-1-ethoxy)benzoyl]-*N*-octanoyl Serinol Trifluoroacetate (**16**) 

Following the general procedure, a solution of **10** (41.4 mg, 0.08 mmol) was treated with TFA (0.2 mL) to afford 38 mg (86%) of **16** as an amorphous white solid .^1^H NMR (400 MHz, DMSO-*d*_6_) δ 8.48 (bs, 18H, NH_3_^+^), 7.36 (s, 4H, Ar), 4.56 (m, 1H, *CH*NH), 4.46–4.23 (m, 16H, CH_2_O), 3.56–3.15 (m, 12H, CH_2_N), 2.16 (t, *J* = 7.3 Hz, 2H, CH_2_CO), 1.47 (m, 2H, CH_2_), 1.17 (m, 8H, CH_2_), 0.80 (t, *J* = 6.4 Hz, 3H, CH_3_). ^13^C NMR (100 MHz, DMSO-*d*_6_) δ 170.0 (CONH), 156.8 (Ar), 145.0 (Ar), 130.2 (Ar), 113.6 (Ar), 74.6 (CH_2_O), 71.0 (CH_2_O), 69.2 (CH_2_O), 51.9 (CHNH), 43.5 (CH_2_N), 40.7 (CH_2_N), 36.2, 33.6, 33.6, 30.5, 27.1 (CH_2_), 19.0 (CH_3_). HPLC (t_R_) [gradient: A:B, 10–100% of A in 10 min]: 2.13 min. HRMS (ESI^+^) *m*/*z*: Calc. for C_37_H_61_N_7_O_11_ 780.4556. Found 779.4425.

#### 4.1.18. Bis-*O*-[3,4,5-tris(2-ammonium-1-ethoxy)benzoyl]-*N*-dodecanoyl Serinol Chloride (**17**) 

Following the general procedure, a solution of **11** (112.5 mg, 0.08 mmol) was treated with TFA (0.4 mL) and co-evaporated several times with HCl 1M in methanol to afford 59.4 mg (50%) of **17** as an amorphous dark yellow solid. ^1^H NMR (400 MHz, DMSO-*d*_6_) δ 8.45 (bs, 18H, NH_3_^+^), 7.36 (s, 4H, Ar), 4.55 (m, 1H, *CH*NH), 4.40 (m, 2H, CH_2_O), 4.36 (m, 2H, CH_2_O), 4.33–4.23 (m, 12H, CH_2_O), 3.33 (m, 8H, CH_2_N), 3.18 (m, 4H, CH_2_N), 2.16 (t, *J* = 7.4 Hz, 2H, CH_2_CO), 1.46 (m, 2H, CH_2_), 1.19 (m, 16H, CH_2_), 0.84 (t, *J* = 6.4 Hz, 3H, CH_3_). ^13^C NMR (100 MHz, DMSO-*d*_6_) δ 173.1 (CONH), 165.3 (COO), 151.7 (Ar), 140.6 (Ar), 125.3 (Ar), 108.6 (Ar), 69.1 (CH_2_O), 65.8 (CH_2_O), 63.7 (CH_2_O), 46.7 (CHNH), 40.68 (CH_2_N), 35.4 (CH_2_CO), 31.3 (CH_2_), 29.1, 28.9, 28.8, 28.6, 25.3, 22.1 (CH_2_), 14.0 (CH_3_). HPLC (t_R_) [gradient: A:B, 10–100% of A in 10 min]: 5.26 min. HRMS (ESI^+^) *m*/*z*: Calc. for C_41_H_69_N_7_O_11_ 836.5152. Found 835.5097.

#### 4.1.19. Bis-*O*-[3,4,5-tris(2-ammonium-1-ethoxy)benzoyl]-*N*-hexadecanoyl Serinol Chloride (**18**) 

Following the general procedure, a solution of **12** (55 mg, 0.03 mmol) was treated with TFA (0.2 mL) and co-evaporated several times with HCl 1M in methanol to afford 27.3 mg (45%) of **18** as an amorphous dark yellow solid. ^1^H NMR (400 MHz, DMSO-*d*_6_) δ 8.41 (bs, 18H, NH_3_^+^), 7.32 (s, 4H, Ar), 4.56 (m, 1H, *CH*NH), 4.42 (m, 2H, CH_2_O), 4.35–4.20 (m, 14H, CH_2_O), 3.34 (m, 8H, CH_2_N), 3.16 (m, 4H, CH_2_N), 2.15 (t, *J* = 7.4 Hz, 2H, CH_2_CO), 1.44 (m, 2H, CH_2_), 1.19 (m, 24H, CH_2_), 0.85 (t, *J* = 6.5 Hz, 3H, CH_3_). ^13^C NMR (100 MHz, DMSO-*d*_6_) δ 172.6 (CONH), 164.8 (COO), 151.7 (Ar), 140.5 (Ar), 125.3 (Ar), 108.6 (Ar), 69.2 (CH_2_O), 65.9 (CH_2_O), 63.8 (CH_2_O), 46.7 (CHNH), 40.7 (CH_2_N), 35.4 (CH_2_CO), 31.2 (CH_2_), 29.1, 29.0, 28.9, 28.8, 28.7, 28.6, 25.4, 22.1 (CH_2_), 14.0 (CH_3_). HPLC (t_R_) [gradient: A:B, 10–100% of A in 10 min]: 6.52 min. HRMS (ESI^+^) *m*/*z*: Calc. for C_45_H_77_N_7_O_11_ 892.5803. Found 891.5714.

#### 4.1.20. Bis-*O*-[3,4,5-tris(2-ammonium-1-ethoxy)benzoyl]-*N*-octadecanoyl Serinol Trifluoroacetate (**19**) 

Following the general procedure, a solution of **13** (50.4 mg, 0.03 mmol) was treated with TFA (0.2 mL) to afford 37 mg (69.6%) of **19** as an amorphous white solid. ^1^H NMR (400 MHz, DMSO-*d*_6_) δ 8.17 (bs, 18H, NH_3_^+^), 7.33 (s, 4H, Ar), 4.58 (m, 1H, CHNH), 4.42 (dd, *J* = 11.2, 5.3 Hz, 2H, CH_2_O), 4.31 (dd, *J* = 11.2, 6.8 Hz, 2H, CH_2_O), 4.25 (t, *J* = 5.1 Hz, 8H, CH_2_O), 4.15 (t, *J* = 5.1 Hz, 4H, CH_2_O), 3.34 (m, 8H, CH_2_N), 3.19 (t, *J* = 4.7 Hz, 4H, CH_2_N), 2.10 (t, *J* = 7.3 Hz, 2H, CH_2_CO), 1.45 (m, 2H, CH_2_), 1.19 (m, 28H, CH_2_), 0.84 (t, *J* = 6.4 Hz, 3H, CH_3_). ^13^C NMR (100 MHz, DMSO-*d*_6_) δ 172.7 (CONH), 164.8 (COO), 151.7 (Ar), 140.4 (Ar), 125.4 (Ar), 108.4 (Ar), 69.4 (CH_2_O), 65.8 (CH_2_O), 63.7 (CH_2_O), 46.8 (CHNH), 40.7 (CH_2_N), 35.5 (CH_2_CO), 31.3, 29.0, 28.9, 28.8, 28.5, 25.3, 22.1 (CH_2_), 14.0 (CH_3_). HPLC (t_R_) [gradient: A:B, 10–100% of A in 10 min]: 7.06 min. HRMS (ESI^+^) *m*/*z*: Calc. for C_47_H_81_N_7_O_11_ 920.6101. Found 919.6006.

#### 4.1.21. Bis-*O*-[3,4,5-tris(2-ammonium-1-ethoxy)benzoyl]-*N*-oleoyl Serinol Trifluoroacetate (**20**) 

Following the general procedure, a solution of **14** (65.1 mg, 0.08 mmol) was treated with TFA (0.4 mL) to afford 65.8 mg (95%) of **20** as an amorphous white solid.^1^H NMR (400 MHz, DMSO-*d*_6_) δ 8.16 (bs, 18H, NH_3_^+^), 7.31 (s, 4H, Ar), 5.27 (m, 2H, CH=), 4.56 (m, 1H, CHNH), 4.39 (m, 2H, CH_2_O), 4.29 (m, 2H, CH_2_O), 4.23 (t, *J* = 5.0 Hz, 8H, CH_2_O), 4.13 (t, *J* = 5.0 Hz, 4H, CH_2_O), 3.32 (m, 12H, CH_2_N), 2.08 (t, *J* = 7.4 Hz, 2H, CH_2_CO), 1.91 (m, 4H, CH_2_CH=), 1.43 (m, 2H, CH_2_), 1.17 (m, 20H, CH_2_), 0.82 (t, *J* = 6.4 Hz, 3H, CH_3_). ^13^C NMR (100 MHz, DMSO-*d*_6_) δ 172.6 (CONH), 164.8 (COO), 151.7 (Ar), 140.6 (Ar), 129.6 (CH=), 130.0 (CH=), 125.2 (Ar), 108.6 (Ar), 69.2 (CH_2_O), 65.9 (CH_2_O), 63.7 (CH_2_O), 46.8 (CHNH), 40.7 (CH_2_N), 35.2 (CH_2_CO), 31.3, 29.5, 29.3, 29.1, 29.0, 28.9, 27.0, 26.6, 25.3, 22.1 (CH_2_), 13.9 (CH_3_). HPLC (t_R_) [gradient: A:B, 10–100% of A in 10 min]: 6.74 min. HRMS (ESI^+^) *m*/*z*: Calc. for C_47_H_79_N_7_O_11_ 918.5827. Found 917.5874.

#### 4.1.22. Bis-*O*-[3,4,5-tris(2-ammonium-1-ethoxy)benzoyl]-*N*-octadecanoyl Serinol Trifluoroacetate (**21**) 

Following the general procedure, a solution of **15** (50 mg, 0.03 mmol) in CH_2_Cl_2_ (10 mL), was treated with TFA (0.25 mL) to afford 26.3 mg (50%) of **21** as an amorphous solid of cream color. ^1^H NMR (400 MHz, DMSO-*d*_6_) δ 8.20 (bs, 18H, NH), 7.34 (s, 4H, Ar), 5.31 (m, 8H, CH=), 4.58 (m, 1H, *CH*NH), 4.43 (m, 2H, CH_2_O), 4.33 (m, 2H, CH_2_O), 4.25 (m, 8H, CH_2_O), 4.17 (m, 4H, CH_2_O), 3.32 (m, 8H, CH_2_N), 3.19 (m, 4H, CH_2_N), 2.75 (m, 4H, CH_2_CH=), 2.65 (m, 4H, CH_2_CH=), 2.13 (m, 2H, CH_2_CO) 1.96 (m, 4H, CH_2_CH=), 1.49 (m, 2H, CH_2_), 1.25 (m, 6H, CH_2_), 0.85 (t, *J* = 8.0, 3H, CH_3_). ^13^C NMR (100 MHz, DMSO-*d*_6_) δ 172.9 (CONH), 166.2 (COO), 152.1 (Ar), 140.8 (Ar), 130.4 (CH=), 129.7 (CH=), 128.6 (CH=), 128.5 (CH=), 128.3 (CH=), 128.2 (CH=), 128.0 (CH=), 127.9 (CH=), 125.8 (Ar), 108.8 (Ar), 69.8 (CH_2_O), 66.3 (CH_2_O), 64.3 (CH_2_O), 47.2 (CHNH), 40.7 (CH_2_N), 35.4 (CH_2_CO), 31.3, 29.1, 28.2, 27.0, 26.6, 25.7, 25.6, 25.5, 22.4 (CH_2_), 14.3 (CH_3_). HPLC (t_R_) [gradient: A:B, 10–100% of A in 10 min]: 6.65 min. HRMS (ESI^+^) *m*/*z*: Calc. for C_49_H_77_N_7_O_11_ 939.5735. Found 938.6043.

### 4.2. Biological Methods

#### 4.2.1. Bacterial Strains

The bacterial strains used in this study included: *Salmonella enterica* serovar Typhimurium SV5015 [34] *Listeria monocytogenes* EGD-e [35], *Staphylococcus aureus* strain Newman [36]. *Staphylococcus aureus* SC-1 (gift of Dr. Daniel López, Centro Nacional de Biotecnología, CNB-CSIC, Madrid, Spain) and USA-300 [28] have been reported as multidrug-resistant (MDR) isolates. These bacteria were grown at 37 °C and shaking conditions (150 rpm) in the following nutrient-rich media: Luria-Bertani (LB) medium for *S.* Typhimurium; brain heart infusion (BHI) medium for *L. monocytogenes*; and, trypticase-soy broth (TSB) for *S. aureus*. Under these growth conditions, the overnight cultures reached an optical density (OD_600_) of 2.0–3.0 (*S.* Typhimurium and *L. monocytogenes*) and 10.0–12.0 (*S. aureus*). 

#### 4.2.2. Determination of Minimal Inhibitory Concentrations (MIC) of Aminophenol Compounds 

Overnight cultures were diluted 1:18,000 (*S.* Typhimurium and *L. monocytogenes*) or 1: 60,000 (*S. aureus*) in fresh media. A volume of 50 µL of this culture was further used to inoculate 2 mL of medium containing serial dilutions (1:4) of the aminophenol compound. The aminoglycoside antibiotic kanamycin was tested in parallel as control. Starting concentrations used were 50 µg mL^−1^ for the aminophenol compounds and 150 µg mL^−1^ for kanamycin. After an overnight culture in shaking conditions, minimal inhibitory concentration (MIC) was determined as the minimum inhibitory concentration of the compound inhibiting bacterial proliferation (lack of turbidity in the culture). 

#### 4.2.3. Monitoring of Bacterial Growth in the Presence of Aminophenol Compounds

Overnight cultures were diluted 1:100 (*L. monocytogenes*) or 1:500 (*S. aureus*) in fresh medium. After aprox. 1.5–2 h of incubation when cultures reached an OD_600_ ~0.2, bacteria were exposed to four-fold the MIC value of the specific antimicrobial to be tested. OD_600_ where then measured every 30 min.

#### 4.2.4. Toxicity Assays in Eukaryotic Cell Cultures

Cultures of normal rat fibroblasts NRK-49F (ATCC CRL-1570) and HeLa human epithelial cells (ATCC CCL-2) were used to assess putative toxicity effects of the aminophenol-derivate compounds. The cells were seeded in 24-well plates and propagated in Dulbecco’s modified Eagle’s medium (DMEM) or Eagle’s minimum essential medium containing 10% (*v*/*v*) fetal bovine serum (FBS) at 37 °C in a 5% CO_2_ atmosphere. Following adherence of the cells to the substrate, the medium was replaced with fresh medium containing different concentrations of the compounds. To 0.5 mL of tissue culture medium per well either 5 µL to 1.25 µL of a 10 mg mL^−1^ stock solution of the compound to test, were added. This was equivalent to a final concentration of 100 to 20 µg mL^−1^, respectively. A control containing 5 µL of the solvent DMSO was analyzed in parallel. The toxicity potential of the compounds was evaluated based on two main parameters: morphological changes in the fibroblast or epithelial cells and fraction of the monolayer culture that lost adherence to the substrate. Changes in these parameters were monitored by microscopy. Cells were fixed with 3% (*v*/*v*) paraformaldehyde (PFA) and processed as described [37]. Images were acquired on an inverted Leica DMI 6000B microscope with an automated CTR/7000 HS controller (Leica Microsystems, Wetzlar, Germany) and an Orca-R2 CCD camera (Hamamatsu Photonics, Hamamatsu, Japan).

#### 4.2.5. Phase-Contrast Microscopy

To determine probable effect of the aminophenol-derivate compounds in bacterial morphology, bacteria were grown to mid-exponential phase (OD600 ~0.2–0.3). At this time, bacteria were exposed for 1 h to a concentration of the compound equivalent to 4 MIC and the OD_600_ measured every 30 min. To monitor probable morphological changes not appreciable by OD_600_, the bacteria were harvested by centrifugation (4300× *g*, 5 min, RT), washed in PBS, fixed with 3% (*v*/*v*) PFA, and processed for microscopy as described [38].

## Figures and Tables

**Table 1:** Antibacterial activity and cytotoxicity of the test compounds.

	MIC ^a^ (µg mL^−1^)					CC ^b^ (µg mL^−1^)
	Drug Sensitive Strains			Multidrug Resistant Strains		
Compound	*S.* Typhimurium SV5015	*L. monocytogenes* EGD-e	*S. aureus* Newman	*S. aureus* SC-1	*S. aureus* USA-300	
**16**	>50 (*)	>50	>50	>50	>50	≥100
**17**	>50	12.5	50	50	12.5	>100 (**)
**18**	>50	3.13	12.5	12.5	3.13	≥100
**19**	>50	3.13	12.5	12.5	50	≥100
**20**	>50	3.13	12.5	12.5	12.5	≥100
**21**	>50	12.5	50	50	12.5	≥100
**Kanamycin**	n/d	2.34	9.4	>150	>150	n/d

(*) no effect in bacterial growth, even at the highest concentration used (50 µg mL^−1^). All values are in µg mL^−1^ and are a summary of multiple dose-response curves (>2) in multiple (>1) experiments. n/d: Not determined. ^a^ MIC: minimum concentration of the compound that inhibits bacterial proliferation after overnight incubation. ^b^ CC: minimum concentration of the compound that inhibits human HeLa epithelial cells or rat fibroblasts NRK-49F proliferation after overnight incubation. (**) In the case of compound **17**, toxicity in NRK-49F fibroblasts was not observed at the highest concentration tested (100 µg mL^−1^).

## Supplementary Materials

Supplementary materials are available online. Copies of representative ^1^H and ^13^C NMR spectra are included.
