# Supplementary material for: A Novel Class of Cationic and Non-Peptidic Small Molecules as Hits for the Development of Antimicrobial Agents
Source: Molecules. 2018 Jun 22;23(7):1513. doi: 10.3390/molecules23071513 (PMC6099707; doi:10.3390/molecules23071513)
Supplement: Supplementary file 1 [file molecules-23-01513-s001.zip › IMAGENES/revised Table 1.docx]

|  | **MIC^a^ (** µg mL^-1^**)** | | | | | **CC^b^**  **(**µg mL^-1^) |
| --- | --- | --- | --- | --- | --- | --- |
|  | Drug sensitive strains | | | Multidrug resistant strains | |  |
| **Compound** | *S.* Typhimurium SV5015 | *L. monocytogenes* EGD-e | *S. aureus*  Newman | *S. aureus*  SC-1 | *S. aureus*  USA-300 |  |
| **16** | >50 (*) | >50 | >50 | >50 | >50 | ≥100 |
| **17** | >50 | 12.5 | 50 | 50 | 12.5 | >100 |
| **18** | >50 | 3.13 | 12.5 | 12.5 | 3.13 | ≥100 |
| **19** | >50 | 3.13 | 12.5 | 12.5 | 50 | ≥100 |
| **20** | >50 | 3.13 | 12.5 | 12.5 | 12.5 | ≥100 |
| **21** | >50 | 12.5 | 50 | 50 | 12.5 | ≥100 |
| **Kanamycin** | n/d | 2.34 | 9.4 | >150 | >150 | n/d |
