# Supplementary figures and images for: A Novel Class of Cationic and Non-Peptidic Small Molecules as Hits for the Development of Antimicrobial Agents
Source: Molecules. 2018 Jun 22;23(7):1513. doi: 10.3390/molecules23071513 (PMC6099707; doi:10.3390/molecules23071513)

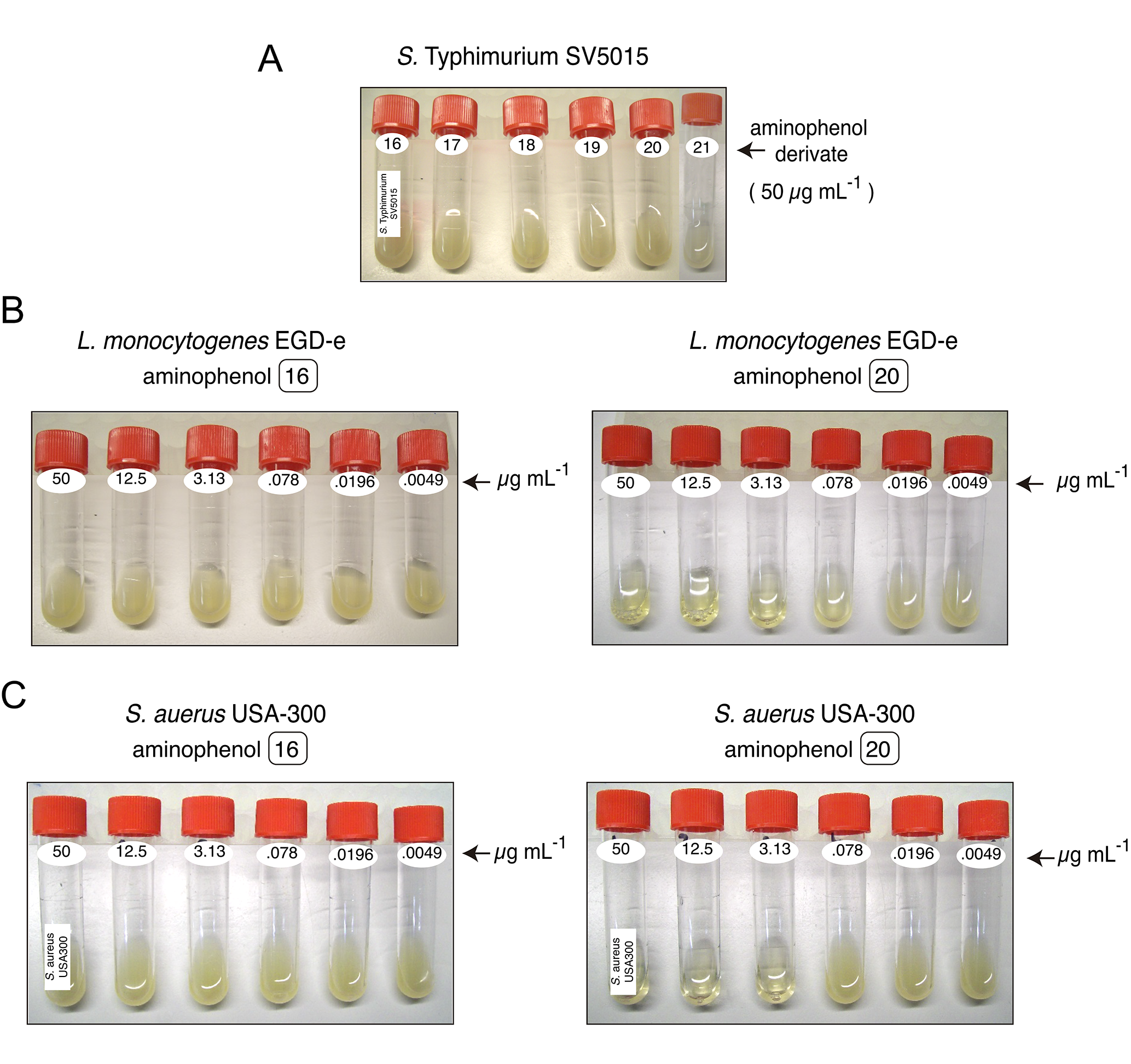

Supplement: Supplementary file 1 [file molecules-23-01513-s001.zip › IMAGENES/FIG-2-revised.tif]

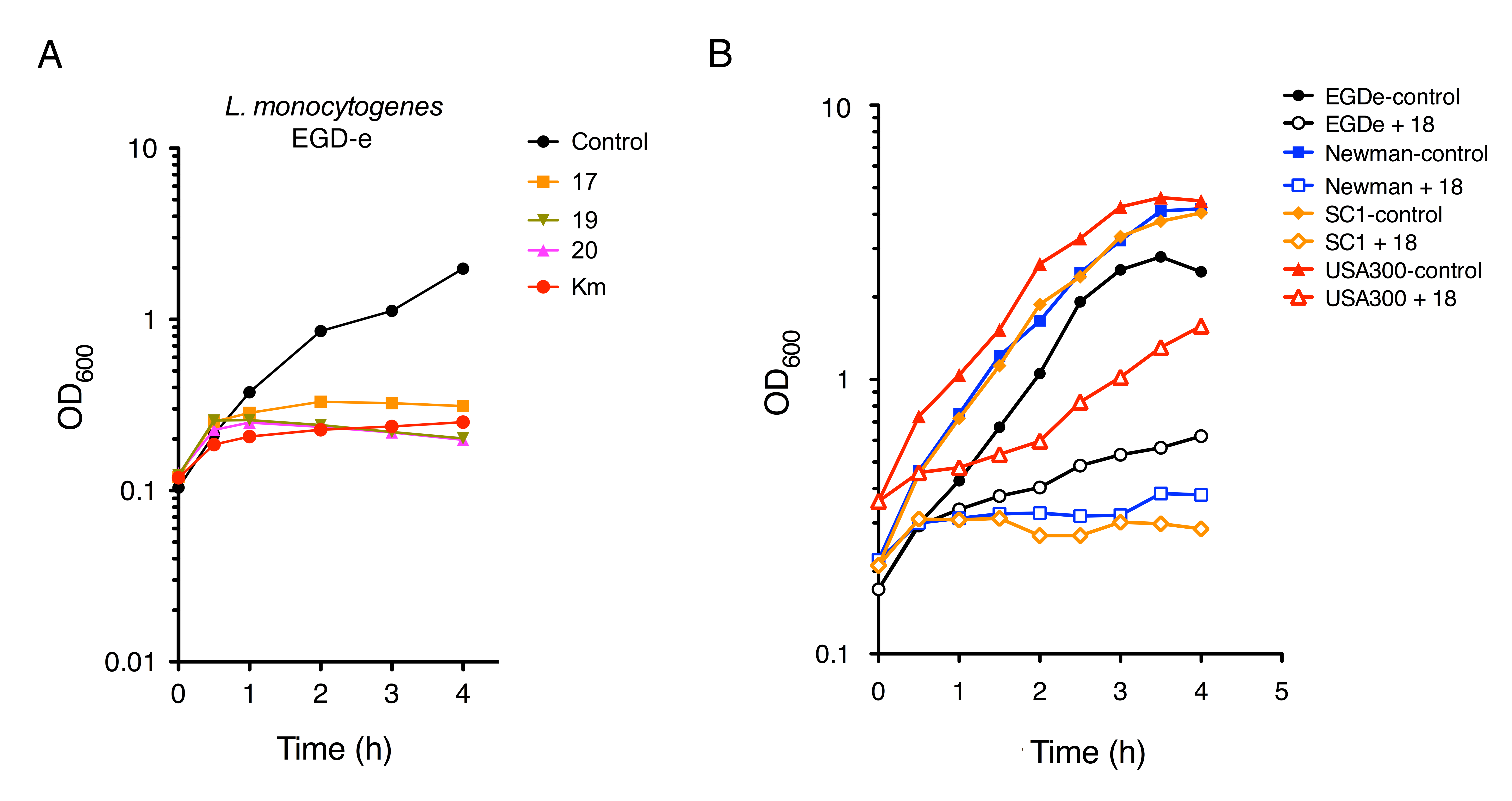

Supplement: Supplementary file 1 [file molecules-23-01513-s001.zip › IMAGENES/FIG-3.tif]

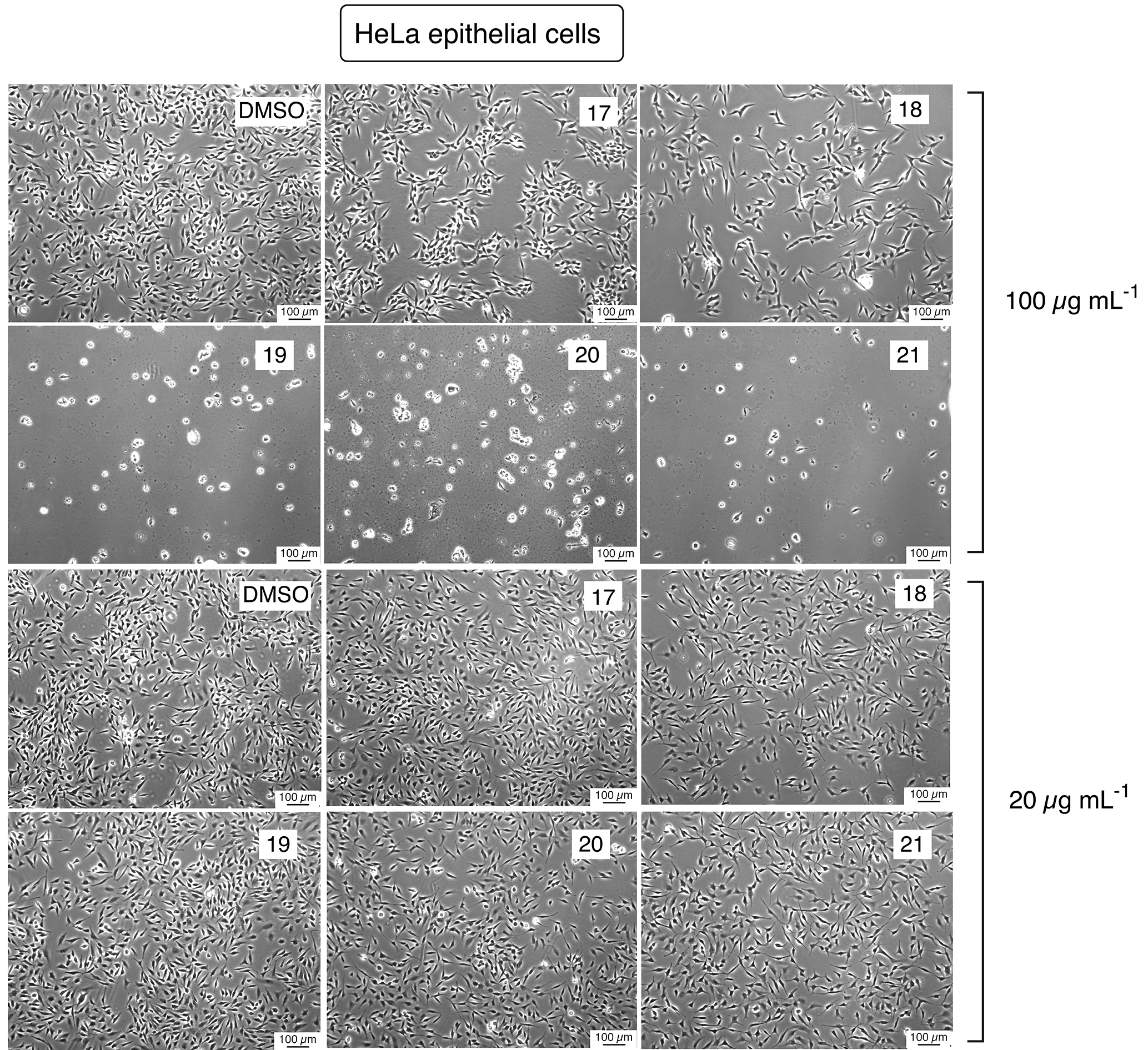

Supplement: Supplementary file 1 [file molecules-23-01513-s001.zip › IMAGENES/FIG-4-revised.tif]

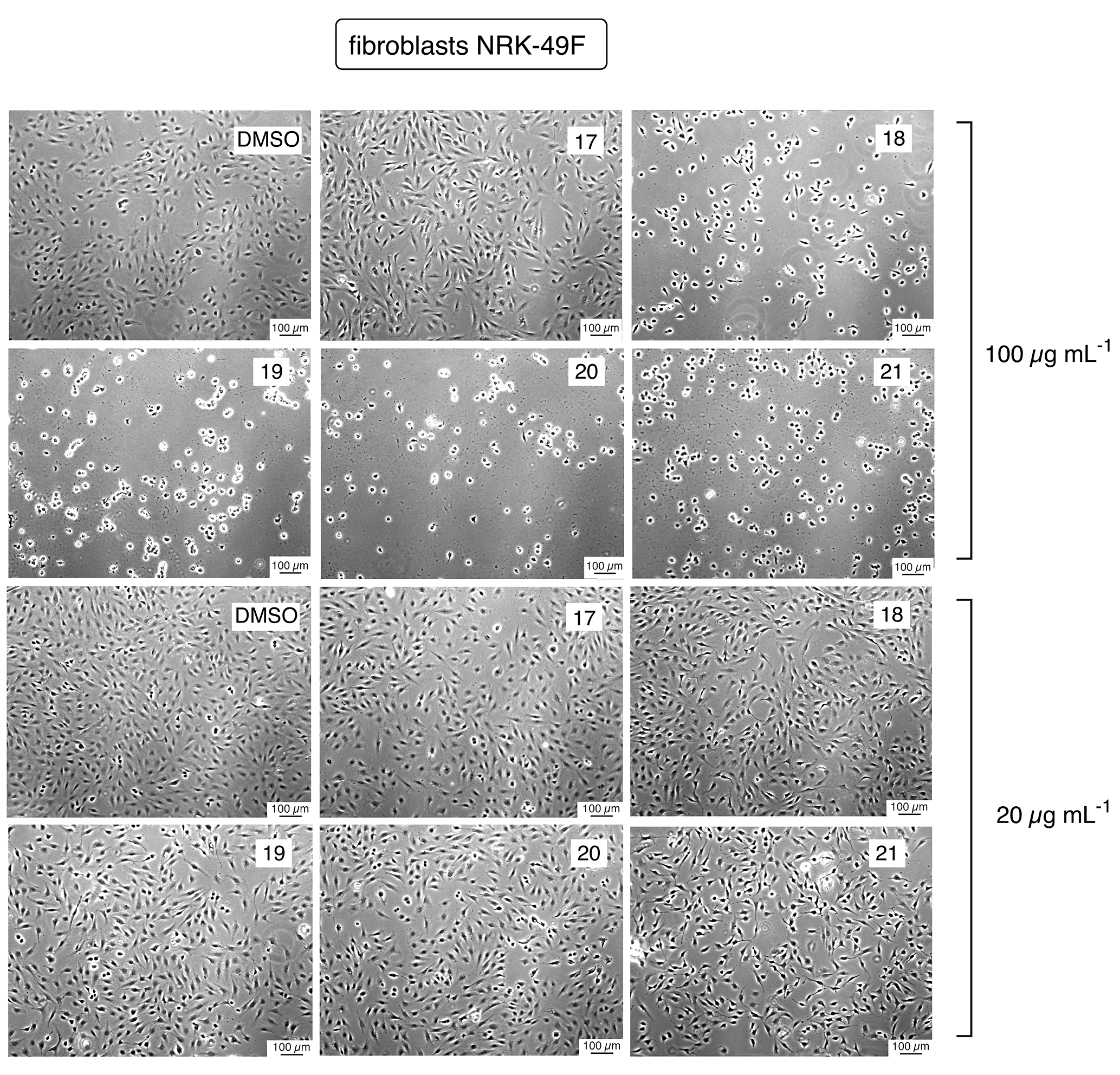

Supplement: Supplementary file 1 [file molecules-23-01513-s001.zip › IMAGENES/FIG-5-revised.tif]
